# Grafting alleviates potassium stress and improves growth in tobacco

**DOI:** 10.1186/s12870-019-1706-1

**Published:** 2019-04-08

**Authors:** Wei Hu, Qing Di, Zhijin Wang, Yimo Zhang, Jie Zhang, Jia Liu, Xiaojun Shi

**Affiliations:** 1 College of Resources and Environment, Southwest University, Chongqing, 400716 China; 2 Vegetable and Flower Institute of Chongqing Academy of Agricultural Sciences, Chongqing, 401329 China; 3 Nanchang Institute of Technology, Nanchang, 330099 China; 4 Soil and Fertilizer & Resources and Environment Institute, Jiangxi Academy of Agricultural Sciences, Nanchang, 330200 China

**Keywords:** Tobacco, Grafting, Potassium, Genes, Root, X-ray microanalysis

## Abstract

**Background:**

Potassium is a nutrient element necessary for tobacco growth. Tobacco leaves with high potassium content are elastic and tough, rich in oil. And the same time, potassium can also improve the scent and aromatic value of flue-cured tobacco by regulating the synthesis of aromatic hydrocarbons in leaves.. It is an important quality indicator for flue-cured tobacco. However, the potassium concentration in tobacco leaves in most areas of China is generally lower than the global standard for high quality tobacco. Two tobacco genotypes were grafted to each other under different potassium levels to test whether potassium content and plant growth can be improved by grafting in tobacco.

**Results:**

The growth of tobacco in all treatments was inhibited under potassium starvation, and grafting significantly alleviated this potassium stress in ‘Yunyan 87’. The trends in whole plant K^+^ uptake and K^+^ transfer efficiency to the leaves corresponded to the growth results of the different grafts. The nutrient depletion test results showed that the roots of ‘Wufeng No.2’ had higher K^+^ absorption potential, K^+^ affinity, and K^+^ inward flow rate. K^+^ enrichment circles appeared at the endoderm of the root section in the energy dispersive X-ray figure, indicating that the formation of Casparian strips may be partly responsible for the lower rate of lateral movement of K^+^ in the roots of ‘Yunyan 87’. Gene expression analysis suggested that energy redistribution at the whole plant level might constitute one strategy for coping with potassium starvation. The feedback regulation effects between scion ‘Wufeng No.2’ and rootstock ‘Yunyan 87’ indicated that the transmission of certain signaling substances had occurred during grafting.

**Conclusions:**

‘Wufeng No.2’ tobacco rootstock grafting can increase the K^+^ uptake and transport efficiency of ‘Yunyan 87’ and enhance plant growth under potassium stress. The physiological mechanism of the improved performance of grafted tobacco is related to higher K^+^ uptake and utilization ability, improved xylem K^+^ loading capacity, and up-regulated expression of genes related to energy supply systems.

## Background

Potassium is an essential macronutrient for the growth of flue-cured tobacco. It is very active in plants and plays important roles in the energy and material conversion of tobacco metabolism [1]. The processes of osmoregulation, photosynthesis, enzyme activation, and formation of carbohydrates, nucleic acids, and proteins are associated with potassium [2]. Potassium content is recognized as an important indicator of quality by the cigarette industry. Tobacco plants growing in conditions that allow the plants to accumulate a sufficiently high concentration of potassium give a product with a better flammability, aromatic taste, and processability of tobacco leaves [3]. However, due to complex soil environments and climatic conditions, potassium deficiency is a common problem in China’s major tobacco growing regions, and the potassium concentration in tobacco leaves in these areas is generally lower than the global standard for high quality tobacco [4]. A variety of agronomic measures have been used to rectify the low potassium content in tobacco leaves. The most cost-effective means is to improve the uptake and utilization efficiency of potassium in tobacco [5].

There are two systems for the absorption of K^+^ in plants: ① under a low K^+^ concentration in the rhizosphere (< 0.2 mM), the absorption process is in accordance with the Michaelis-Menten equation, namely a high-affinity transport system (HATS). ② Under a high K^+^ concentration in the rhizosphere (> 1 mM), the K^+^ uptake of plants is linear with the external K^+^ concentration, namely a low-affinity transport system (LATS) [6]. The absorption of K^+^ by HATS is an inverse-electrochemical potential gradient process. It is carried out by H^+^/K^+^ or K^+^/Na^+^ coordinated transportation with potassium transporters. LATS is determined by the free diffusion of ions, which is responsible for the potassium channel and exhibits a high degree of non-specificity for the absorption of K^+^ [7]. The energy for K^+^ active absorption of plants is provided by two supply systems: H^+^-ATPase and H^+^-PPase. *NHA1* and *NVP1* partly regulate the expression of H^+^-ATPase and H^+^-PPase, respectively [8]. Analysis of the gene expression associated with the pathway of potassium transport is helpful for understanding the molecular mechanisms associated with highly efficient potassium utilization genotypes.

Since Epstein applied the enzymatic reaction kinetic equation to study the ion absorption process of roots [9], the kinetic analysis of ion assimilates has been used to evaluate the nutrient uptake efficiency of plants. In many studies, the affinity and absorption capacity of the roots for ions in soil and solution is usually represented by *C*_min_, *K*_m_, and *I*_max_ through the depletion test [10–14]. Therefore, analyzing the differences in potassium absorption kinetics in the roots of different tobacco genotypes can provide a reference for the selection of high potassium tobacco rootstock materials. X-ray microanalysis is used to investigate elemental localization at the cellular level of plants [15]. This technology can be used to analyze the distribution of elements in various tissues of plants and the transportation mode of nutrient ions under different conditions [16, 17].

As a traditional agronomic measure, grafting has been widely used to improve abiotic stress tolerance and nutrient uptake in addition to improving resistance to soil-borne pests and diseases [18, 19]. Studies have indicated that genetic signal transmission and gene drift exist in the process of grafting, which makes the expression of certain genes in the rootstocks affect the characteristics of the scion [20, 21]. Compared with un-grafted controls, the disease symptoms of grafted tobacco infected with tobacco mosaic virus were controlled, which was thought to be related to changes in the miRNA expression level [22]. When the *Arabidopsis* miR399 gene deletion mutant was used as a rootstock material, the wild-type scion miR399 was able to transport from the scion through the phloem to the rootstock, activating the root Pi response gene PH02 [23]. No in-depth research is available regarding the use of grafting to improve the utilization efficiency of potassium in tobacco. Therefore, two tobacco genotype varieties were used as experimental grafting materials to verify whether grafting can increase tobacco performance under potassium starvation.

## Results

### Plant growth

The different grafting combinations and potassium supply treatments significantly impacted plant growth (Table 1). Compared with the +K treatment, the whole plant dry weight, leaf area, and root surface area, length, and volume were significantly decreased under –K treatments. In the case of the +K treatments, the growth parameters of the plant were significantly higher in the grafting combinations of W and Y/W than in W/Y and Y. However, the difference between the two treatments sharing the same rootstock was not significant (such as W and Y/W). With regards to the –K treatments, the plant growth parameters in the grafting combinations of W and Y/W were still significantly higher than those in the W/Y and Y. In addition, the differences between the treatments of W and Y/W were not significant, but the performance of the W/Y treatments was significantly better than the Y treatments, which differed from the results of the +K treatments. Potassium deficiency caused the whole plant dry weight, leaf area, and root surface area, length, and volume of tobacco to decline. Compared with the +K treatments, the -K treatments resulted in a smaller decrease of the whole plant dry weight in W (29.7%) and Y/W (30.1%) compared to W/Y (42.5%) and Y (50.8%). The former did not constitute a statistically significant difference, while the latter achieves significant differences. The trends in leaf area, root surface area, and length and volume of tobacco were similar.

**Table 1:** Effect of graft combination and K^+^ level on tobacco growth

Graft combination	+K					−K				
	whole plant dry weight (g)	leaf area (cm^2^)	root surface area (cm^2^)	root length (cm)	root volume (cm^3^)	whole plant dry weight (g)	leaf area (cm^2^)	root surface area (cm^2^)	root length (cm)	root volume (cm^3^)
W	2.25a	81.42a	21.93a	271.09a	1.42a	1.58a	75.81a	17.79a	243.72a	1.13a
Y/W	2.02a	74.33a	19.82a	253.71a	1.34a	1.41a	69.77a	16.75a	218.98a	1.06a
W/Y	1.42b	56.47b	15.34b	195.57b	1.18b	0.81b	54.79b	12.42b	170.19b	0.82b
Y	1.37b	52.47b	14.19b	182.33b	1.07b	0.55c	41.02c	9.28c	143.33c	0.63c

Different letters within the same column indicate a significant difference (*P* < 0.05)

### Uptake and transport of K^+^ through the roots to the leaves

**Fig. 1:**
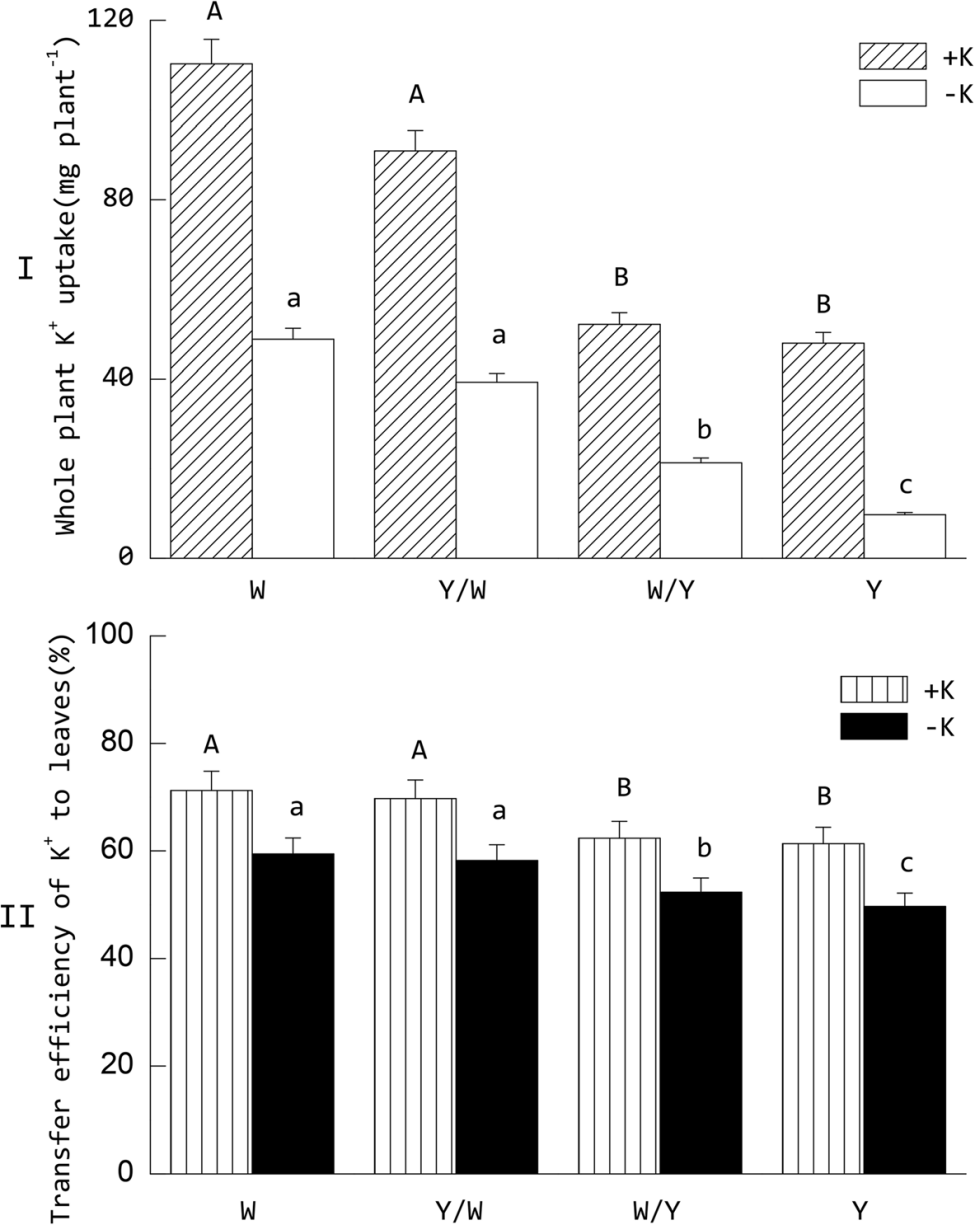
Effect of grafting combination and K^+^ supply level on whole plant K^+^ uptake (I) and transfer efficiency of K^+^ to the leaves (II). The tobacco graft combinations included the ungrafted tobacco W (Wufeng No.2) and Y (Yunyan 87), and grafted tobacco Y/W (Y grafted onto W) and W/Y (W grafted onto Y). Different uppercase letters denote significant differences (*P* < 0.05) under normal potassium levels (5 mmol L^− 1^) and lowercase letters indicate significant differences (*P* < 0.05) under starvation (0.5 mmol L^− 1^)

It was found that the K^+^ absorption and transport efficiency of tobacco was affected by the grafting and potassium supply situation (Fig. 1). Potassium stress simultaneously reduced the capability of whole plant K^+^ uptake and transfer efficiency of K^+^ to the leaves. For the different grafting treatments, the W genotype tobacco was not only superior to the Y genotype in terms of whole plant K^+^ uptake and transfer efficiency of K^+^ to the leaves, but the performance of these indices was also enhanced when Y was used as the rootstock. Under the +K treatments, whole plant K^+^ uptake and the transfer efficiency of K^+^ to the leaves in the treatments using W as the rootstock increased 97.6 and 14.06% compared with those in treatments using Y as the rootstock. Under the –K treatments, these metrics increased to 187.76 and 15.21%, respectively.

### Kinetics of K^+^ absorption in tobacco

**Fig. 2:**
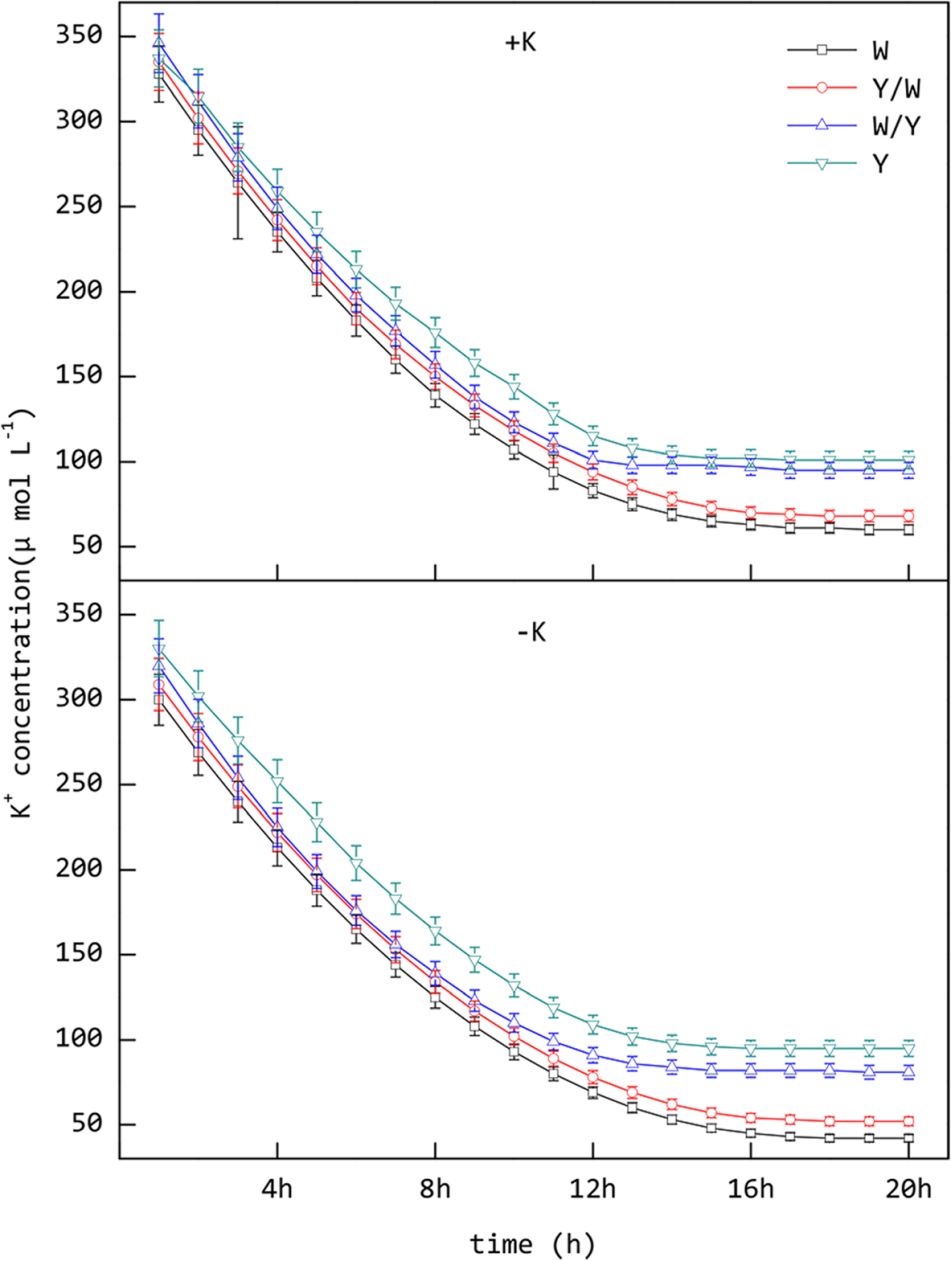
Absorption kinetics curve of K^+^ in tobacco roots under different treatments. +K represents normal potassium levels (5 mmol L^− 1^) and -K represents potassium starvation (0.5 mmol L^− 1^). Ungrafted tobacco W (Wufeng No.2) and Y (Yunyan 87), and grafted tobacco Y/W (Y grafted onto W) and W/Y (W grafted onto Y) during the 20 h nutrient depletion test

Based on the K^+^ concentration curve in the depleted fluid over time, the binomial Eq. (1) could be derived (Fig. 2). The K^+^ absorption kinetic parameter in the roots was then obtained through the calculation. The results of the statistical analysis demonstrated that, in the case of the +K treatments, the differences in *I*_max_, *K*_m_, *C*_min_, and α between W and Y/W were not significant. The same was observed in the W/Y and Y treatments. However, the differences in *I*_max_, *K*_m_, *C*_min_, and α between the treatments of W, Y/W and W/Y, Y were significant (Table 2). This situation illustrates that when the potassium supply is sufficient, the rootstock of genotype W tobacco has better potassium absorption capacity than Y, and the different scion treatments have little effect on this ability. Compared with the +K treatments, the values of the parameter related to K^+^ uptake under starvation were improved in the –K treatments. The *I*_max_ and α in the roots of the W and Y/W plants were significantly higher than those of W/Y, Y, while *K*_m_ and *C*_min_ were significantly lower in the –K treatments. In addition, the differences between the W and Y/W treatments were not significant. It is worth noting that the differences between the W/Y and Y treatments were significant, which differs from the case of the +K treatments. This suggests that using genotype W tobacco as the scion could improve K^+^ absorption in the roots of genotype Y tobacco under K^+^ deficit.

**Table 2:** Absorption kinetics parameters of K^+^ in tobacco roots under different treatments

Graft combination	+K				-K			
	*I*_max_ (μmol FW g^−1^ h^−1^)	*K*_m_ (μmol L^− 1^)	*C*_min_ (μmol L^− 1^)	α	*I*_max_ (μmol FW g^− 1^ h^− 1^)	*K*_m_ (μmol L^− 1^)	*C*_min_ (μmol L^− 1^)	α
W	91.56a	133.17b	57.37b	0.69a	105.21a	113.81c	41.51c	0.92a
Y/W	88.22a	140.71b	65.64b	0.63a	100.57a	121.84c	49.12c	0.83a
W/Y	58.39b	160.07a	87.63a	0.36b	70.4b	142.35b	74.27b	0.49b
Y	55.74b	165.91a	96.84a	0.34b	58.3c	158.99a	90.58a	0.37b

Different letters within the same column indicate a significant difference (*P* < 0.05)

### X-ray microanalysis of the K^+^ distribution in the root tissues

**Fig. 3:**
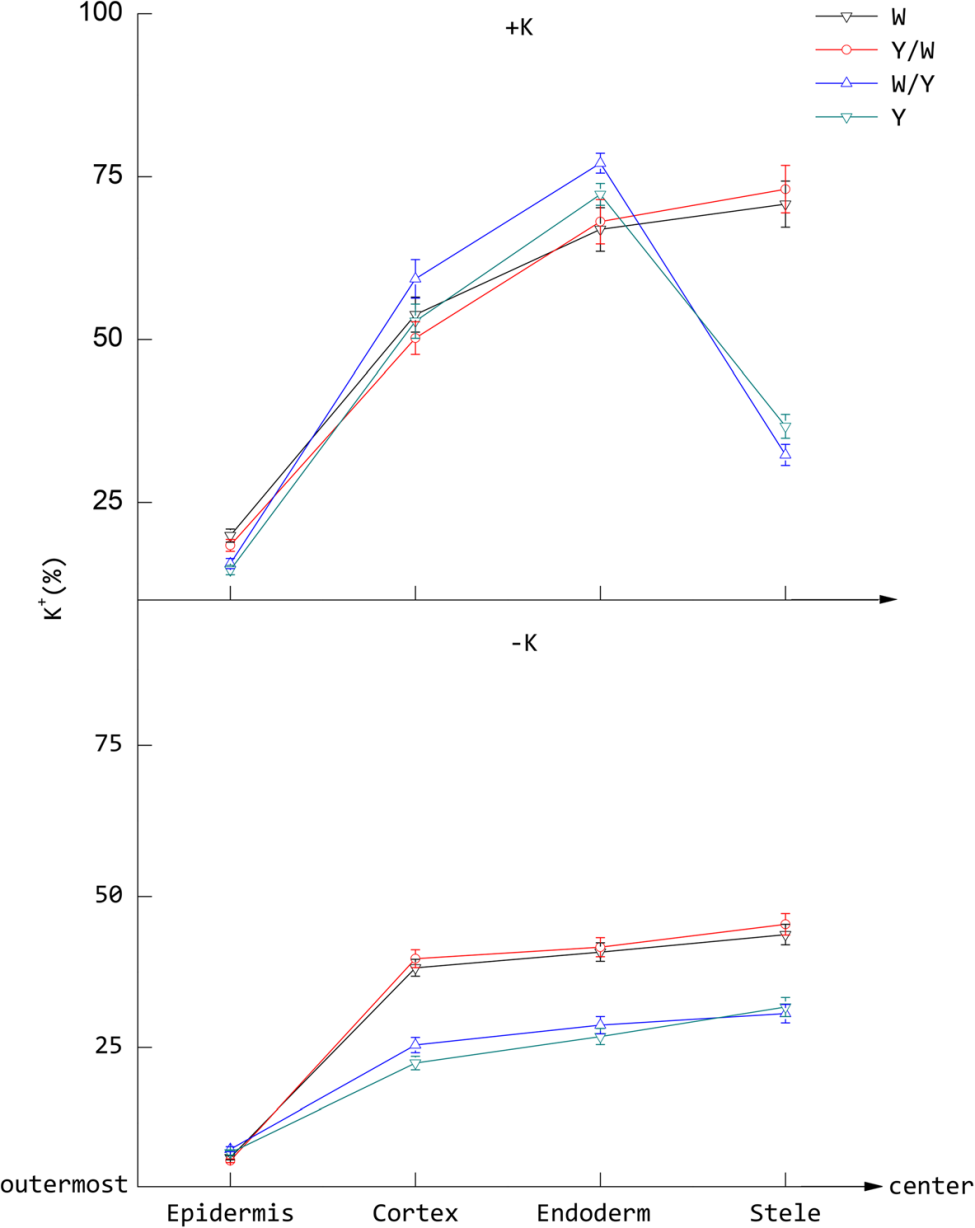
Elemental distribution of K^+^ in various tissues of the tobacco roots under different treatments. Data represent the relative atomic content of K^+^ as a percentage of all test elements (Na^+^, K^+^, Ca^2+^, P, S, Cl^−^, Mg^2+^) in a given region. The tobacco graft combinations included the ungrafted tobacco W (Wufeng No.2) and Y (Yunyan 87), and grafted tobacco Y/W (Y grafted onto W) and W/Y (W grafted onto Y)

**Fig. 4:**
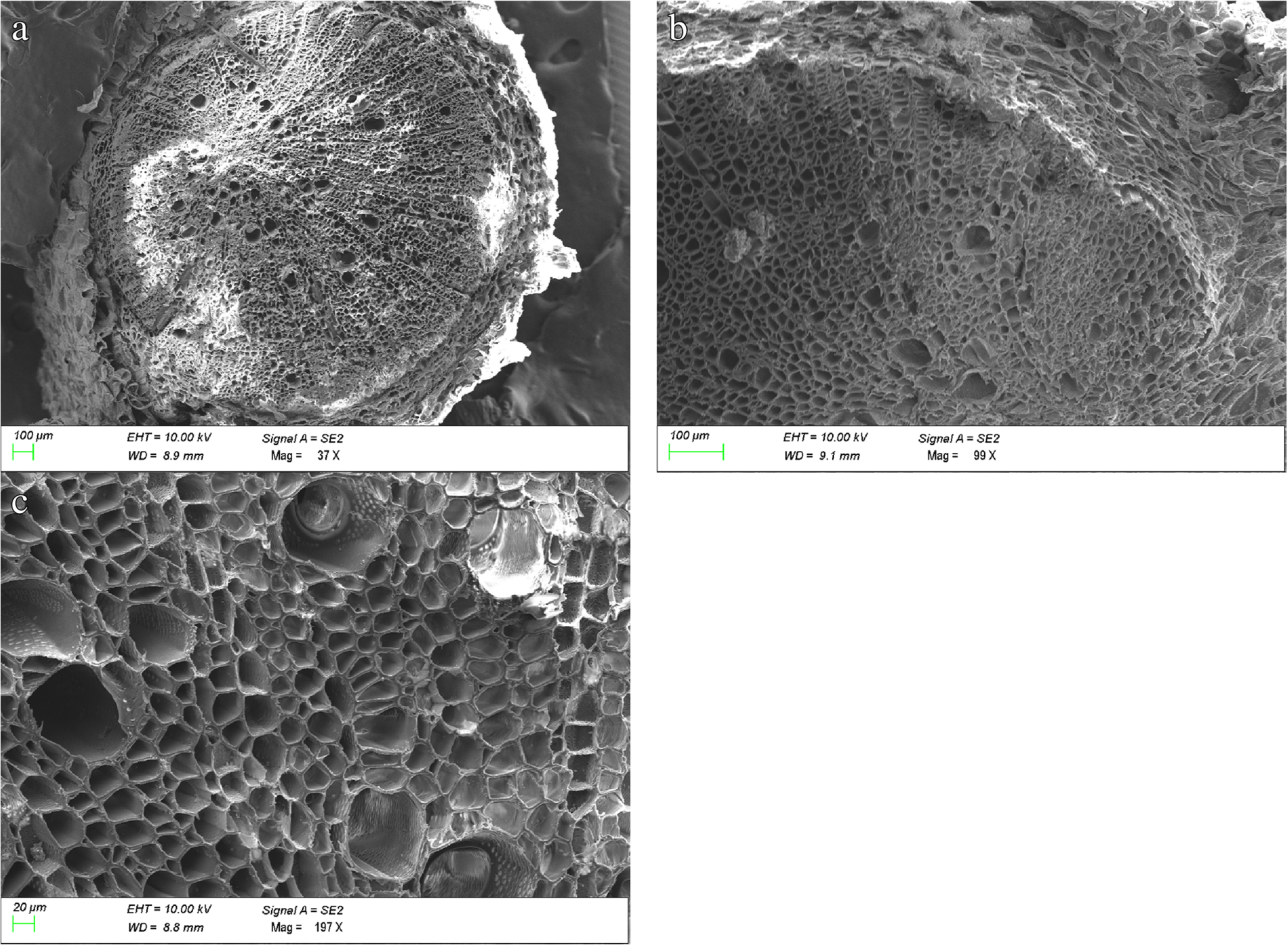
Scanning electron micrographs of a root tissue section under different magnifications. Different lowercase letters represent different magnifications

**Fig. 5:**
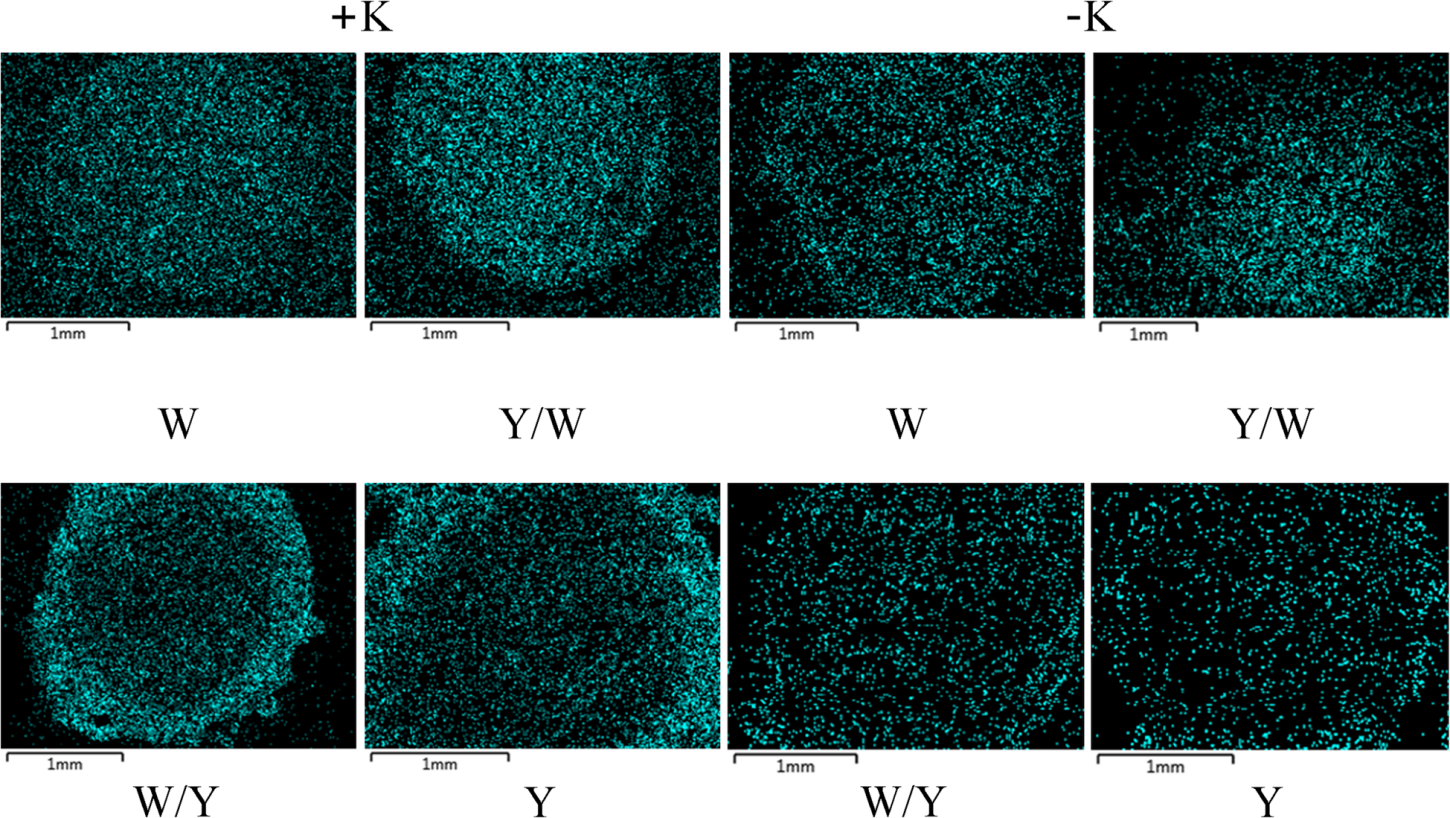
Images of the K^+^ distribution in tobacco roots from the X-ray microanalysis. Blue particles represent the charge of K^+^. The tobacco graft combinations included the ungrafted tobacco W (Wufeng No.2) and Y (Yunyan 87), and grafted tobacco Y/W (Y grafted onto W) and W/Y (W grafted onto Y)

Compared with the –K treatments, there was an obvious advantage in the relative K^+^ content of each tissue of the root transverse sections under the +K treatments. Potassium deficiency decreased the mean relative K^+^ content by 58.1, 44.3, 30.9, and 28.8% in the epidermal cells, cortical cells, endoderm cells, and stelar parenchyma of the roots, respectively (Figs. 3 and 4). In the –K treatments, the relative K^+^ content increased in a gradient from the outer root tissues to the middle tissues for all grafting combinations, and the highest accumulation occurred in the stelar parenchyma. Although the relative K^+^ contents were higher in the grafting combinations of W and Y/W than W/Y and Y, the difference was not statistically significant. When the potassium supply was normal, the difference between the grafting combinations in whole transverse sections with different rootstocks was great. The trend of K^+^ distribution in grafting combinations W and Y/W was similar to the –K treatments. But the relative K^+^ content peaked in the endoderm cells and dramatically decreased in the stelar parenchyma in the grafting combinations of Y and W/Y. For the grafting combinations of Y and W/Y, the relative K^+^ content of the endoderm cells was significantly higher in the +K treatments than –K by 168.6 and 169.8%, respectively. In addition, K^+^ enrichment circles were observed near the endoderm cells in the grafting combinations of Y and W/Y under +K treatments, but did not exist in the grafting combinations of W and Y/W or –K treatments (Fig. 5).

### Effects of grafting on the expression of potassium transport genes

**Fig. 6:**
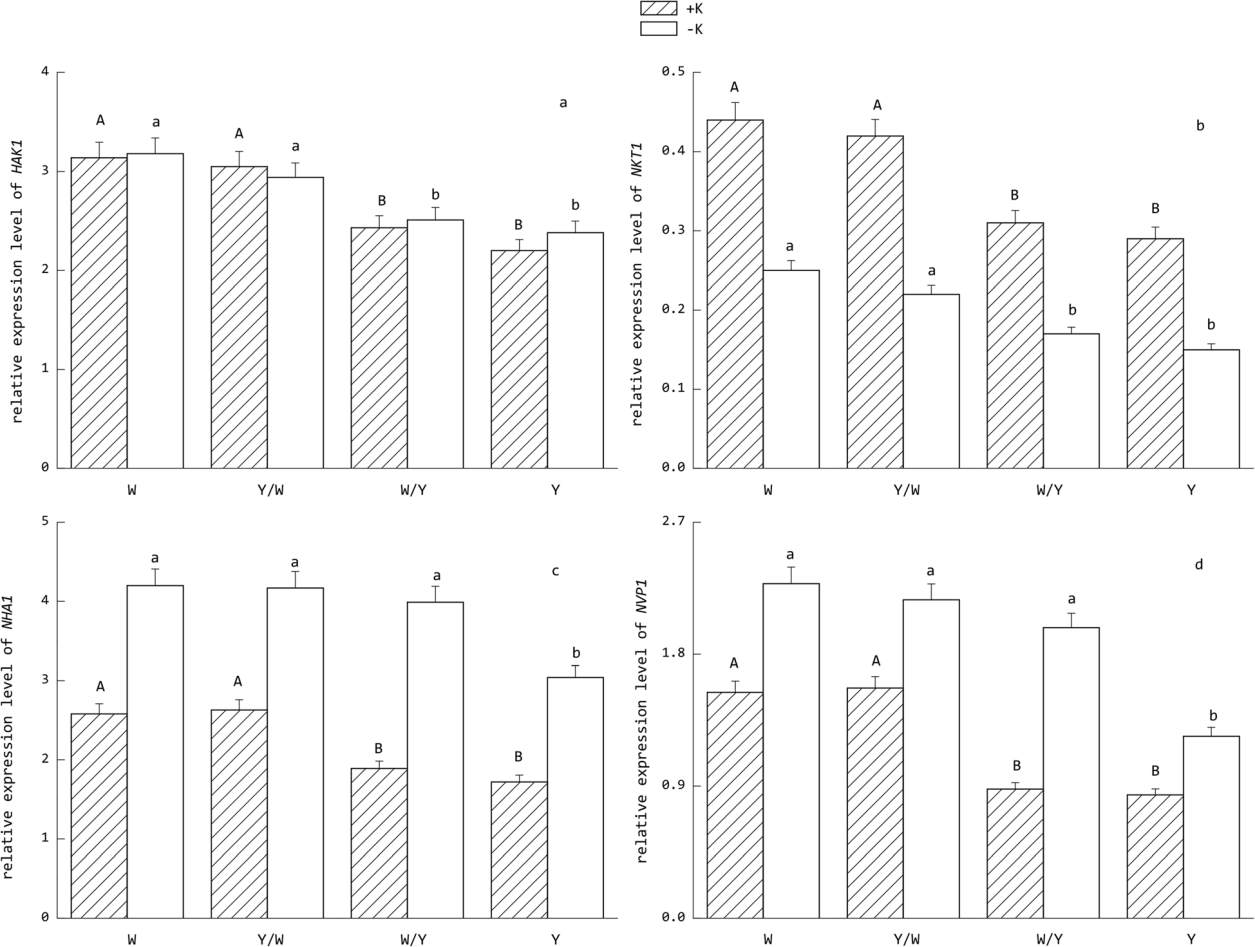
Relative expression levels of genes (a. *HAK1*, b. *NKT1*, c. *NHA1*, d. *NVP1*) related to potassium transport in tobacco roots under two potassium supply conditions. The tobacco graft combinations included the ungrafted tobacco W (Wufeng No.2) and Y (Yunyan 87), and grafted tobacco Y/W (Y grafted onto W) and W/Y (W grafted onto Y). Different uppercase letters denote significant differences (*P* < 0.05) under normal potassium levels (5 mmol L^− 1^) and lowercase letters indicate significant difference (*P* < 0.05) under potassium starvation (0.5 mmol L^− 1^)

Potassium stress significantly altered the expression level of *HAK1*, *NKT1*, *NHA1,* and *NVP1* in the tobacco roots. As shown in Fig. 6, the expression of *HAK1* was not significantly altered under various potassium levels between each grafting combination, while the converse was observed for *NKT1. NHA1* and *NVP1* were up-regulated under the induction of environmental potassium deficient conditions. For the different graft combinations, the expression levels of each gene in the W and Y/W groups were significantly higher than those in the Y and W/Y treatments under normal potassium supply levels. Notably, *NHA1* and *NVP1* in the W/Y treatments were significantly up-regulated under the condition of potassium deficiency, and their expression level was not significantly lower than the W and Y/W grafting combination. As observed for *HAK1*, *NKT1* did not demonstrate this trend, and its expression was still significantly lower than the W and Y/W grafting combinations.

## Discussion

### Grafting improves tobacco plant growth and K^+^ uptake under potassium starvation

Potassium movement in plants is highly positive, which means it is capable of active transmembrane transportation and the activation of multiple enzymatic reactions. As an activator of aminoacyl-tRNA and polypeptide synthesis, potassium deficiency in tobacco plants blocks the synthesis pathway of proteins, resulting in a large accumulation of soluble amino acids in the cells [24]. A previous study suggested that grafting improves tolerance to low potassium stress in watermelon [25], but its efficiency is dependent on rootstock [26]. The result of this trial demonstrated that the plant growth parameters of tobacco obviously decreased under potassium deficiency, but the degree of decline differed in each grafting combination. Compared with the +K treatments, the -K treatments only slightly decreased the whole plant dry weight in W and Y/W than W/Y and Y. The trends in leaf area, and root surface area, length, and volume of tobacco were thus similar. Our results also showed that tobacco exhibits better growth when the W genotype, rather than the Y genotype, is used as the rootstock. At the same time, grafting improved the plant growth performance of W/Y when W was used as a scion even under potassium stress. This indicates that there is a feedback regulation between scion W and rootstock Y. The long-distance signal dominates this feedback regulation mechanism in grafting, and miRNA or hormonal signal may acts as signaling substances [27–29]. However, this feedback regulation only occurred in the W/Y grafting combination, and the Y/W grafting combination did not appear to exhibit this regulation, which indicates that the induction condition of the feedback regulation differed between the two tobacco genotypes.

The potassium content of the leaves, as the economic organ of tobacco, greatly affects its commercial value [3, 30, 31]. Grafting can affect the utilization efficiency of multiple nutrients in tobacco [32–34]. In our study, potassium stress simultaneously reduced the capability of whole plant K^+^ uptake and transfer efficiency of K^+^ to the leaves. Of the different grafting combinations, the W genotype was not only superior to the Y genotype in whole plant K^+^ uptake and transfer efficiency of K^+^ to the leaves, but also significantly enhanced the performance of these indices when grafted onto Y as the rootstock. This result indicates that in addition to the higher absorption and transport capacity of potassium, the treatments using W as rootstocks have better adaptability than Y in response to environmental potassium deficiency stress. Conversely, the grafting combination W/Y exhibited significantly higher whole plant K^+^ uptake and transfer efficiency of K^+^ to the leaves than Y, which indicates that grafting could improve the K^+^ utilization of tobacco.

Root nutrient uptake kinetics is an effective means of elucidating plant nutrient absorption characteristics [10, 11]. The quantification of K^+^ uptake by plants is useful for analyzing the affinity and adaptability of different genotypes of tobacco to potassium. The results of the exhaustion test indicate that the W genotype has better K^+^ absorption potential, affinity, and rate of nutrient influx into the root system and a minimum absorption concentration than the Y genotype. In addition to this, the absorption capacity of K^+^ in the tobacco roots was enhanced by potassium stress according to the experimental data. The feedback regulation effect of the scion on the rootstock was not obvious during normal potassium supply, but became significant under potassium stress conditions and mainly indicated positive adjustment. This phenomenon corroborates the observed differences in the growth of each treatment. Previous studies have shown that there is a long-distance feedback regulation of scion on rootstock in potassium absorption [35]. But the specific signal substance has not been fully confirmed [36].

### An endodermis barrier decreased K^+^ transport efficiency from the outer root tissues to the middle tissues

The data in this study show that the difference in transport efficiency of K^+^ between the roots of the two genotypes is not related to the ability of K^+^ to enter the epidermis and cortex. It was mainly associated with the loading capacity of K^+^ to the stelar parenchyma. This is similar to the findings of Lei [37] with regards to salt stress in pumpkin. The emergence of K^+^ enrichment circles in Y and W/Y under normal potassium supply indicates that the K^+^ transportation barrier in the endodermal cells may partly explain why the Y genotype was weaker than the W genotype with regards to potassium transport in the roots. Further, the K^+^ content of the stelar parenchyma in the roots of the W varieties was higher than that of the Y varieties, whereas the cortex and endoderm cells had lower K^+^ than the Y varieties. This indicated that the W genotype had higher xylem K^+^ loading capacity and could improve the transport efficiency of K^+^ from the root to the shoot under normal potassium supply. Under potassium deficient conditions, the difference in K^+^ distribution in the root tissues between the W and Y genotype was not significant. This indicates that the increased K^+^ absorption and transport in the W genotype under the absence of potassium was derived from the shoot rather than the root system. It also accounts for the significant difference between the grafting combination W/Y and Y in terms of plant growth and the uptake and transport of K^+^ through the root to the leaf. Specific cell types in designated locations are required for the management of K^+^ movement within plants. In contrast to water migration, solutes such as Na^+^, K^+^, Ca^2+^, and abscisic acid (ABA) move freely in the apoplast of the roots and need to pass the Casparian strips of the endodermis to reach the apoplast of the xylem [38–40]. The asymmetric distribution of K^+^ under different potassium levels in Y and W/Y could be attributed to the barrier and filtering functions of the Casparian strips.

### Analysis of gene expression related to the potassium transport pathway

The uptake and transport of potassium in plants is regulated by multiple gene families, including the K^+^ channel family [41], high affinity transporter family [42], co-transporter family [43], reverse transporter family, and proton pump genes for energy supply [44]. *HAK1* belongs to the family of high-affinity K^+^ transporters and is believed to be involved with the absorption of low K^+^ concentrations in the rhizosphere [45, 46]. Among the four *Shaker-like* K^+^ channel genes expressed in tobacco cells, *NKT1* represents an inwardly rectifying K^+^ channel that mediates K^+^ uptake [47]. *NHA1* and *NVP1* partly regulate the expression of H^+^-ATPase and H^+^-PPase, respectively, the former being located at the cytoplasmic membrane, while the latter is located at the tonoplast. Their function is to drive the K^+^ secondary co-transport system by electrochemical potential difference [48]. In this study, the responses of the four genes differed greatly in each treatment under potassium starvation. The expression of *HAK1* was not significantly altered under various potassium levels in each grafting combination, which indicates that it was not induced under a low K^+^ environment. This result is consistent with the conclusions of Ahn et al. [49] who found that expression of KT/KUP gene in Arabidopsis is not induced by low environmental K^+^ levels. Down-regulated expression of *NKT1* and up-regulated expression of *NHA1* and *NVP1* suggests that energy redistribution at the whole plant level may be one of the strategies for coping with potassium starvation by reducing unnecessary consumption and activating more efficient potassium transport pathways [50]. For the different tobacco genotypes, the expression levels of each gene in W were significantly higher than in Y. This may explain their different performances in K^+^ absorption and transport in tobacco roots. In addition to this, using W genotype tobacco as the scion increased the expression level of *NHA1* and *NVP1* in the roots for the Y genotype. This indicates that the up-regulated expression of genes related to the energy supply of transmembrane transport is important for the response of tobacco to potassium deficiency. Moreover, this regulation can be transmitted from the scion to the rootstock via signaling substances. Plants consume energy in response to stress and in the defense response. However, the negative effect of the expression of defense genes on the growth of plants sometimes exceeds the positive effects [51]. Resistance to nutrient stress is often accompanied by the inhibition of plant growth [52]. Some defense gene knockout *Arabidopsis* mutants even show higher growth rates than the wild-type in the early growth period [53]. Thus, the down-regulated expression of *NKT1* and up-regulated expression of *NHA1* and *NVP1* were more conducive to the growth of tobacco under low potassium stress conditions. Furthermore, different tissues and organs of tobacco exhibit different gene expression patterns related to the pathway of K^+^ transport [54], which requires further investigation.

## Conclusion

In conclusion, ‘Wufeng No.2’ tobacco rootstock grafting can increase the K^+^ uptake and transport efficiency of ‘Yunyan 87’ and enhance plant growth under potassium stress. The physiological mechanism of the improved performance of grafted tobacco is related to higher K^+^ uptake and utilization ability, better xylem K^+^ loading capacity, and up-regulated expression of genes related to energy supply systems. The feedback regulation effects between scion W and rootstock Y indicated that the transmission of certain signaling substances had appeared during grafting.

Compared with the common tobacco cultivar ‘Yunyan 87’, ‘Wufeng No.2’ exhibits obvious advantages in the absorption and transport of potassium. However, a higher nicotine content and lower accumulation of aromatic precursor substances in baked tobacco leaves renders ‘Wufeng No.2’ less favorable than other raw tobacco leaves in industrial production. This research shows that the Y/W grafting combination not only preserves the advantages of the ‘Wufeng No.2’ rootstock in potassium absorption and transport, but also inherits the excellent characteristics of the scion of ‘Yunyan 87’ in terms of leaf nicotine content and the accumulation of aromatic precursor substances (unpublished data from this research project team). These findings have the potential to greatly increase the value of ‘Yunyan 87’ as an industrial raw material.

## Methods

### Plant materials and treatments

The trial was carried out in the greenhouse of the Chongqing Academy of Agricultural Sciences, Southwest China (latitude 29°36′N, longitude 106°29′E, and altitude 290 m above sea level). The flue-cured tobacco of the K efficient genotype ‘Wufeng No.2’ (*Nicotiana tabacum*, Yichang Tobacco Company of Hubei Province, China) and common cultivar ‘Yunyan 87’ (*N. tabacum*, Yunnan Tobacco Research Institute, China) were used in this study. Tobacco seeds were surface-disinfected in 0.1% HgCl_2_ for 10 min and soaked in sterile water for 15 min, after which they were rinsed three times. The seeds of the rootstocks were sowed into 100-cell plug trays (filled with peat, vermiculite, and puffed perlite at a volume ratio of 3: 1: 1) and the seeds of the scion were sowed 7 d later. Grafting was carried out when the seedlings of the rootstock had developed six to eight true leaves by ‘split grafting’. Silicone tubes and graft clips were used depending on the thickness of the rootstocks and scions during grafting. After 3–5 days of incubation and moisturizing in the graft healing room, the seedlings were transferred to the general shed. When the grafted plants had produced new leaves, they were transplanted into a 25-L plastic containers for hydroponic cultivation (the inner dimensions of the length, width, and height were 43 cm, 27 cm, and 24 cm, respectively), with 12 seedlings planted per container. The hydroponic nutrient solution, a special formula for flue-cured tobacco seedlings, was formulated as follows: NO_3_-N 8 mmol L^− 1^, PO_4_-P 1 mmol L^− 1^, K 6 mmol L^− 1^, Ca 4 mmol L^− 1^, Mg 2 mmol L^− 1^, Fe 0.09 mmol L^− 1^, Mn 9 μmol L^− 1^, Cu 0.3 μmol L^− 1^, Zn 0.8 μmol L^− 1^, B 46.25 μmol L^− 1^, and Mo 0.5 μmol L^− 1^. In order to ensure oxygen supply to the tobacco seedlings in the experiment, air pumps were used to supply oxygen to each container through a hose. The plants were grown at 22.5 °C under a 16-h light/8-h dark cycle using fluorescent lamps with an average photosynthetic photon flux density (PPFD) of 300 μmol m^− 2^ s^− 1^ in the greenhouse. The relative humidity ranged from 60 to 95%.

There were two potassium levels in this trial: +K, normal concentration (5 mmol L^− 1^) and -K, starvation (0.5 mmol L^− 1^), using K_2_SO_4_ as the substance source. The other macronutrients and micronutrients were the same in the solutions. In addition, four graft combinations [Wufeng No.2 (W), Yunyan 87 (Y), W/Y (W grafted onto Y), Y/W (Y grafted onto W)] were adopted in this experiment. The eight treatments were replicated six times with eight plants in each replicate and were arranged in a randomized complete block design. The solutions were renewed every 4 days to meet the nutrient requirements for the growth of the tobacco seedlings. The plant samples were collected 20 d after low potassium treatment.

### Determination of plant growth

Four plants per treatment were used to determine dry matter weight, leaf area, and root morphology. The second last true leaf on the stem of the tobacco seedlings was chosen as the sample for leaf area measurement using a leaf area meter (LI-3000C, LI-COR, USA). The sample plants were divided into three parts as the root, stem, and leaf(shoot was the part above graft union and root was the part below graft union. For ungrafted plants, the part above the cotyledon node was regarded as the “shoot”, and the part below was the “root”). The dry weight of the root, stem, and leaf was measured using an electronic balance after drying for 24 h at 70 °C in a forced air oven. The roots were rinsed with deionized water, and excess water was blotted carefully with tissue paper. The root surface area, length, and volume were analyzed by WinRhizo Pro software. Potassium content in the dried samples was determined using a flame atomic absorption spectrometer (Varian AA-220FS, Thermo Fisher Scientific, USA) as previously described [55]. The calculation of whole plant K^+^ uptake and transfer efficiency of K^+^ to the leaves was as follows:

$$ \mathrm{Whole}\ \mathrm{plant}\ {\mathrm{K}}^{+}\mathrm{uptake}=\left(\mathrm{root}\ \mathrm{dry}\ \mathrm{weight}\ \mathrm{root}\kern0.5em \times \kern0.5em {\mathrm{K}}^{+}\mathrm{concentration}\right)+\left(\mathrm{stem}\ \mathrm{dry}\ \mathrm{weight}\kern0.5em \times \mathrm{stem}\ {\mathrm{K}}^{+}\mathrm{concentration}\right)+\left(\mathrm{leaf}\ \mathrm{dry}\ \mathrm{weight}\times \mathrm{leaf}\ {\mathrm{K}}^{+}\mathrm{concentration}\right)\mathrm{Transfer}\ \mathrm{efficiency}\ \mathrm{of}\ {\mathrm{K}}^{+}\mathrm{to}\ \mathrm{leaves}=\frac{\mathrm{leaf}\kern0.5em \mathrm{dry}\ \mathrm{weight}\kern0.5em \times \kern0.5em \mathrm{leaf}\ {\mathrm{K}}^{+}\mathrm{concentration}}{\mathrm{Whole}\kern0.5em \mathrm{plant}\kern0.5em {\mathrm{K}}^{+}\mathrm{uptake}}\times 100\% $$

### Kinetics of K^+^ absorption in the roots

Four plants exhibiting similar growth in each treatment were used for the depletion test [56]. Before the start of the trial, the tobacco seedlings were placed in a K-free nutrient solution for 48 h, the pH was adjusted to 6.0, and the solution was changed once a day during the potassium starvation treatment. After that, the plants were transferred to a flask filled with 400 mL of depletion fluid, made up of 0.2 mmol L^− 1^ K_2_SO_4_ in darkness. One milliliter of depletion fluid was used for the determination of K^+^ concentration and was replenished with 1 mL of deionized water at the same time to ensure that the volume of depleted fluid was constant. Samples were taken once an hour for 20 h. At the end of the depletion test, the fresh root weight (FRW) was calculated after the moisture was removed with filter paper. The flask was ventilated during the entire depletion test. The potassium concentration in the depletion fluid was measured using a continuous flow method [57]. When the rate of absorption of K^+^ was at half the maximum rate of K^+^ absorption, the concentration in the solution is the Michaelis-Menten constant (*K*_m_). The maximum rate of K^+^ absorption is *I*_max_. When the K^+^ absorption rate was zero, the K^+^ concentration in the nutrient solution is *C*_min_. In order to calculate *K*_m_ and *I*_max_, the ion exhaustion curve equation must be established. The commonly used one-variable twice polynomial is as follows:

1 $$ Y=a+ bx+{cx}^2, $$

where *x* represents the absorption time and *Y* represents the K^+^ concentration in the depletion fluid at that time. To obtain the first derivative of *x* from (I), derive the equation as follows:

2 $$ {Y}^{'}=2 ax+b, $$

where *Y′* represents the ion absorption rate. For Eq. (2), let *x* → 0, *Y′* = |*b*|. Substituting the *b* value into Eq. (3), the *I*_max_ can be known. *V* represents the volume of the depleting fluid.

3 $$ {I}_{max}\left(\upmu \mathrm{mol}\ \mathrm{FW}\ {\mathrm{g}}^{\hbox{-} 1}{\mathrm{h}}^{\hbox{-} 1}\right)=\frac{b\times V}{FRW\left(\mathrm{g}\right)} $$

For Eq. (2), let *Y′* = 1/2|*b*| to find *x*, then substitute it into Eq. (1) to find *Y*, following which the *K*_m_ can be known. Similarly, let *Y′* = 0 for *C*_min_ to be known. In order to compare the rate of ion flow into the root system between each treatment, the parameter α was defined as the nutrient inflow rate. The formula is as shown:

4 $$ \upalpha ={I}_{\mathrm{m}\mathrm{ax}}/{K}_{\mathrm{m}} $$

### X-ray microanalysis of transverse root sections in tobacco

Samples were processed as indicated in Peng et al. [58], with modifications based on the specifications of the tobacco roots. The seedling roots were washed with distilled water three times, and transverse sections (0.1 cm thickness) of the roots were made between 1 and 2 cm from the root tip by free-hand sectioning with a razor blade. Following this, the sections were placed into a centrifuge tube and snap-frozen in liquid nitrogen. In order to remove all the moisture from the samples, the sections were freeze-dried for 120 h using a lyophilizer (SJIA-10 N-50A, Shuangjia, China). The samples were gold plated in a high vacuum sputter coater and stored in a desiccator. Energy dispersive X-ray microanalytical detection was carried out under standardized conditions, as described in Bucking and Heyser [59], using a scanning electron microscope (Sigma 300, Zeiss, Germany) equipped with an energy dispersive spectrometer (INCA, Oxford, UK). Probe measurements of the roots were taken with a broad electron beam to analyze the relative K^+^ levels within the epidermal cells, cortical cells, endoderm cells, and stelar parenchyma of the roots under different treatments. Three transverse sections of each treatment were observed and three location spots of the same tissue of each section were analyzed. The distribution of K^+^ in the various tissues of the root transverse sections was determined by calculating the area of the absorption peak in Aztec One software. A relative amount of K^+^ was expressed as a percentage of the total atomic number for all major elements (Na^+^, K^+^, Ca^2+^, P, S, Cl^−^, Mg^2+^) detected from the root tissues.

### Total RNA extraction and quantitative real-time PCR

Total RNA was isolated from roots of each treatment with TRIzol reagent (Invitrogen, ThermoFisher, USA) following the manufacturer’s protocol. DNA contaminants were removed by RNase-free DNase. First-strand cDNA synthesis was performed in a 20 μL reaction system using 1 μg of total RNA in combination with oligo (dT)-18 as a primer and M-MuLV reverse transcriptase (TaRaKa, Japan). Expression of the target genes was measured by quantitative real-time PCR (ABI 7900HT, Applied Biosystems, USA) following the instructions of the LightCycler480 SYBR Green I Master kit. The thermal cycling conditions comprised an initial step at 95 °C for 15 s, followed by 40 cycles at 95 °C for 10 s, 60 °C for 20 s, and 72 °C for 15 s. Each sample analysis was repeated at least three times. DNAMAN 8.0 software was used for the design of gene-specific primers, and the primers used are summarized in Table 3. The melting curve analysis showed that each primer present high specificity. The PCR products were quantified by the LightCycler480 real-time PCR detection system with a SYBR Green I master kit, and the data were analyzed using the 2^−ΔΔCt^ method [60].

**Table 3:** Primers used in the qRT-PCR analysis

Gene	Accession Number	Forward primer (5′-3′)	Reverse primer (5′-3′)
*HAK1*	DQ841950	ATCCACACCGAGCTTGTTTCAGGA	TGGGTCCAATTCTTCCCACCAAGA
*NKT1*	AB196790	TTGCTGGTGATGGTACTTCAG	TACCCGCCCTAGATTAGTCG
*NHA1*	AY383599	GGAAGTTCGACTTCTCGCCA	CACAGCTAGCCCTTGTTCTT
*NVP1*	X83730	GGCACGTAACCACAGTGAAG	AGAAGCAGCCATGCCTAGTC
Actin^a^	AB158612	AACAGTTTGGTTGGAGTTCTGG	CATGAAGATTAAAGGCGGAGTG

^a^reference gene (Act) for qRT-PCR analysis

### Statistical analysis

The data in the tables and figures are expressed as the means of all replicates ± standard deviation (S.D.) Data were statistically analyzed by analysis of variance (ANOVA) using SAS Version 9.3 (Statistical Analysis System Institute Inc., Cary, NC, USA). Statistical significance was assessed using the least significant difference (LSD) test (*P* < 0.05).

## Abbreviations

*HAK1*: *Nicotiana tabacum* high-affinity potassium transporter protein 1
*NHA1*: *Nicotiana tabacum* proton P-ATPase mRNA
*NKT1*: *Nicotiana tabacum* potassium channel protein 1
*NVP1*: *Nicotiana tabacum* mRNA for inorganic pyrophosphatase
qPCR: quantitative real-time PCR
